# Supplementary material for: Hierarchical Porous P-Doped NiCo Alloy with α/ε Phase-Defect Synergy to Boost Alkaline HER Kinetics and Bifunctional Activity
Source: Nanomaterials (Basel). 2025 Oct 14;15(20):1562. doi: 10.3390/nano15201562 (PMC12566926; doi:10.3390/nano15201562)
Supplement: Supplementary file 1 [file nanomaterials-15-01562-s001.zip › nanomaterials-3917150-supplementary.pdf]

Hierarchically Porous P-Doped NiCo Alloy with α/ε Phase-Defect Synergy to Boost Alkaline HER Kinetics and Bifunctional Activity

Lun Yang,[#] Meng Zhang,[#] Mengran Shi,[#] Yi Yao, Ying Liu, Jianqing Zhou, Yi Cao, Zhong Li, Meifeng Liu, Xiuzhang Wang, Zhixing Gan, Haixiao Zhang, Shuai Chang, Gang Zhou,[*] Yun Shan[*]

Table of content

Fig. S1. SEM images of hierarchical porous Ni (hp-Ni).
Fig. S2. SEM images of hierarchical porous Co (hp-Co).
Fig. S3. SEM images of bare Ni foam (NF).
Fig. S4. SEM images and EDS elemental mapping of hp-NiCo.
Fig. S5. SEM images and EDS elemental mapping of P-NiCo@Cu Foam.
Fig. S6. SEM images of phosphorus-doped NiCo (P-NiCo).
Fig. S7. SEM image of phosphorus-doped hierarchical porous NiCo (hpP-NiCo).
Fig. S8. SEM image, EDS elemental mapping, and EDS spectrum of hpP-NiCo.
Fig. S9. TEM image and EDS elemental mapping of hp-NiCo.
Fig. S10. TEM image and EDS elemental mapping of hpP-NiCo.
Fig. S11. SEM images of phosphorus-doped Ni (P-Ni).
Fig. S12. SEM images of phosphorus-doped Co (P-Co).
Fig. S13. XRD patterns of samples deposited on Ni foam (NF) substrate.
Fig. S14. XRD standard patterns (PDF cards) for α/ε-NiCo and α-Ni/ε-Co.
Fig. S15. XPS survey spectra of hp-NiCo, P-NiCo, and hpP-NiCo.
Fig. S16. HER polarization & Tafel plots of NF, P-Ni, P-Co, P-NiCo.
Fig. S17. HER current density comparison: hpP-NiCo vs. reported Ni/Co catalysts.
Fig. S18. OER polarization & Tafel plots of NF, P-Ni, P-Co, P-NiCo.
Fig. S19. CV curves for capacitive current measurement of NF, P-NiCo, and hp-NiCo.
Fig. S20. CV curves of P-NiCo, hp-NiCo, and hpP-NiCo in 1 M PBS.
Fig. S21. N_2 adsorption–desorption & pore size distribution of hpP-NiCo (BET).
Fig. S22. EIS Bode magnitude plots of hpP-NiCo.

Table S1. Performance comparison of various HER catalyst (η_{100} (mV))

Table S2. Performance comparison of various HER catalyst (j_{150} (mA cm^{-2}))

Table S3. Equivalent circuit fitting data of hpP-NiCo.

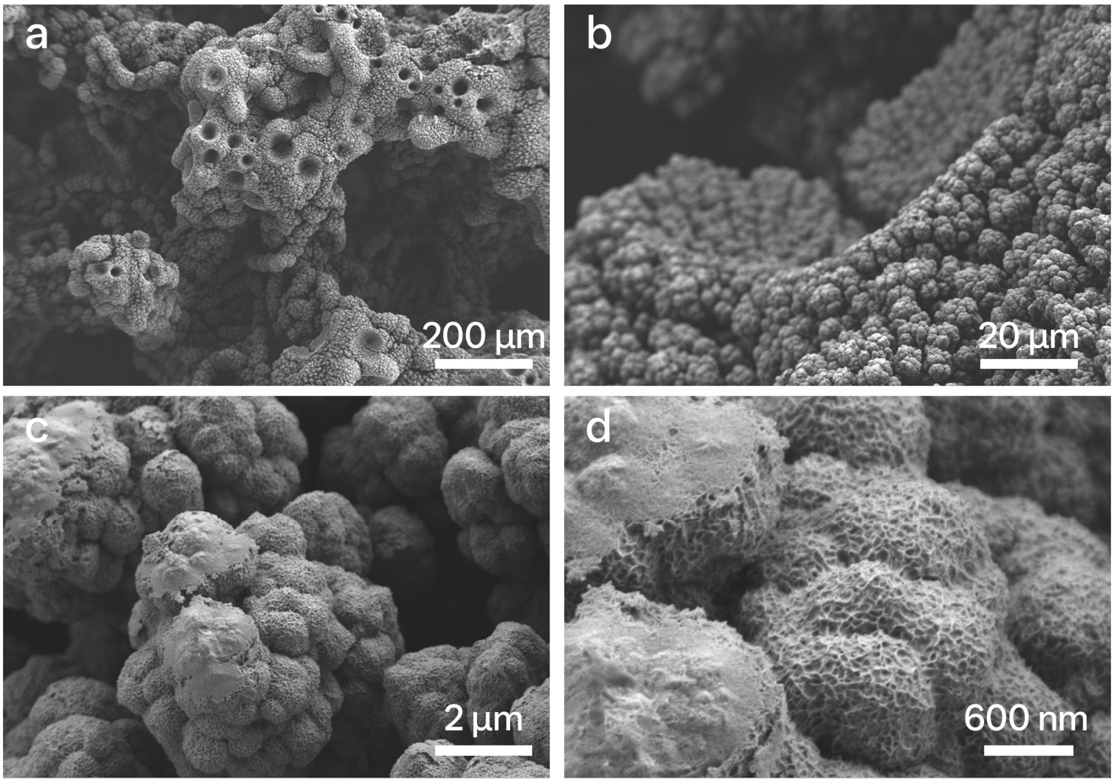

Figure S1. (a–d) SEM images of hierarchical porous Ni (hp-Ni) synthesized via the hydrogen bubble template method, showcasing the multiscale porous architecture.

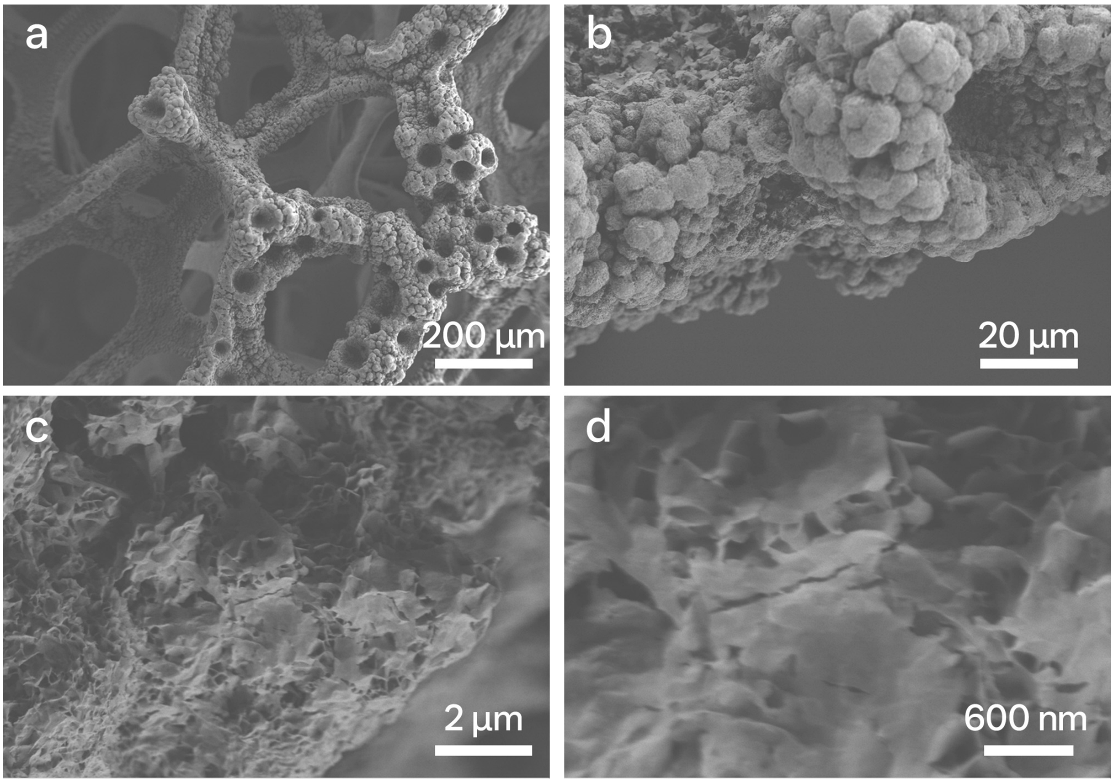

Figure S2. (a–d) SEM images of hierarchical porous Co (hp-Co) fabricated via the hydrogen bubble template method, illustrating the hierarchical porous structure.

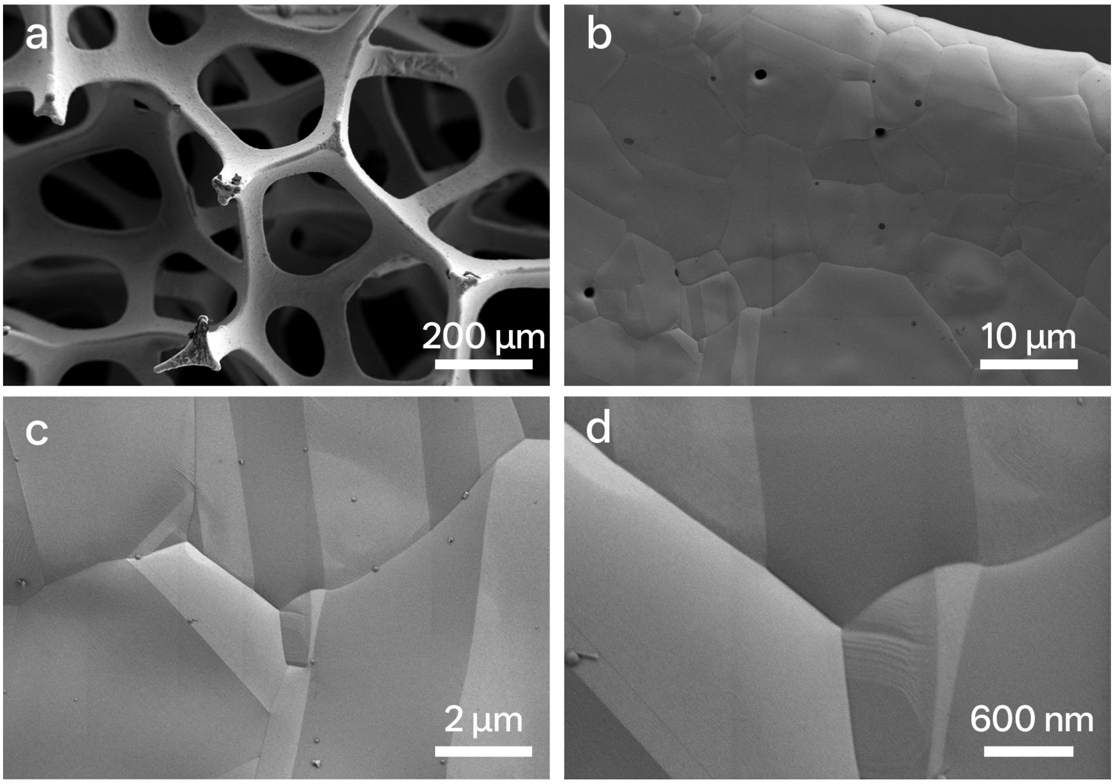

Figure S3. (a–d) SEM images of bare nickel foam (NF), showing its three-dimensional porous architecture and intrinsic surface features at increasing magnifications.

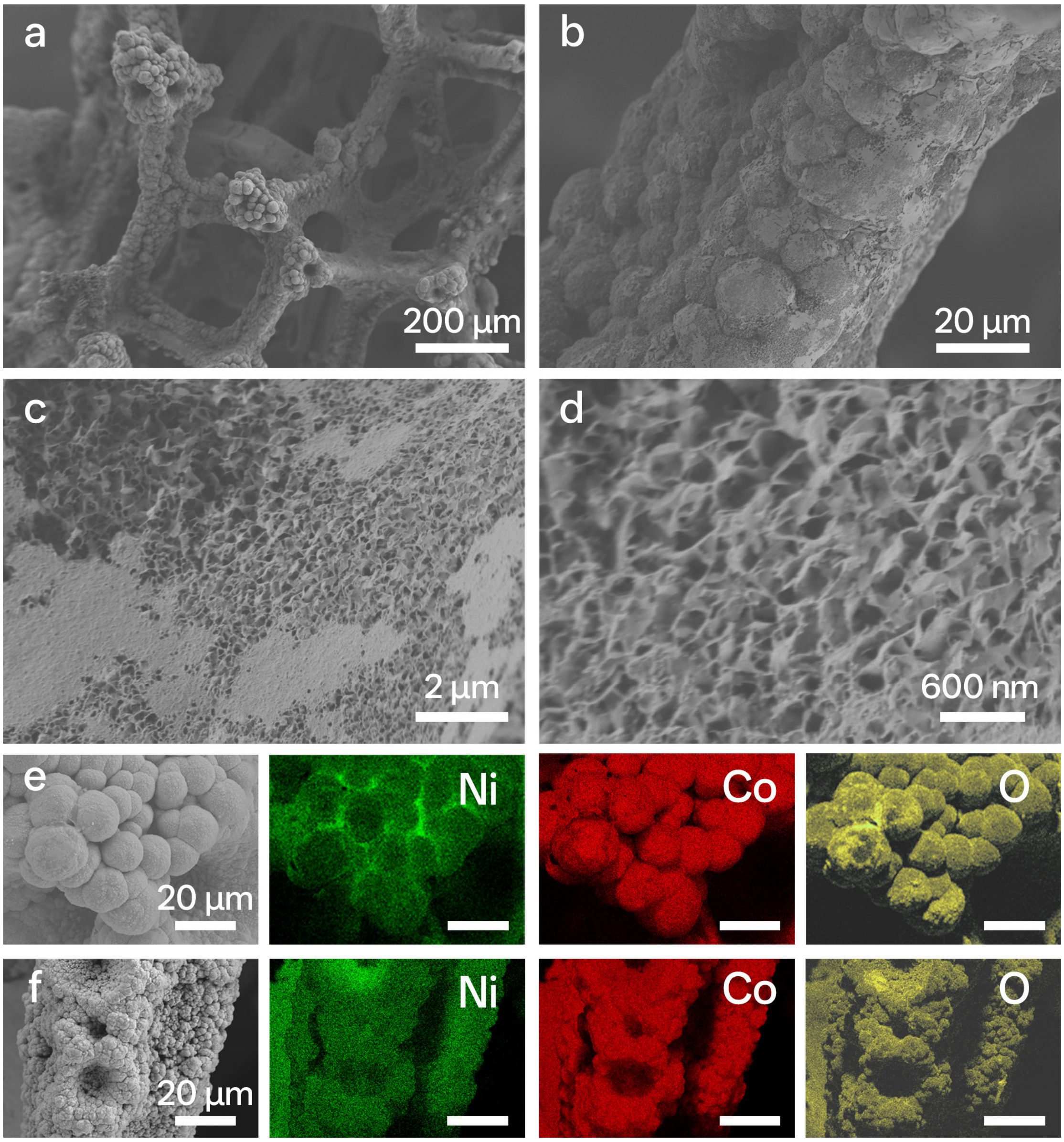

Figure S4. (a–d) SEM images of hierarchical porous NiCo (hp-NiCo) prepared via the hydrogen bubble template method, demonstrating the 3D hierarchical porous framework. (e–f) Corresponding EDS elemental maps (Ni, Co, O) revealing homogeneous element distribution.

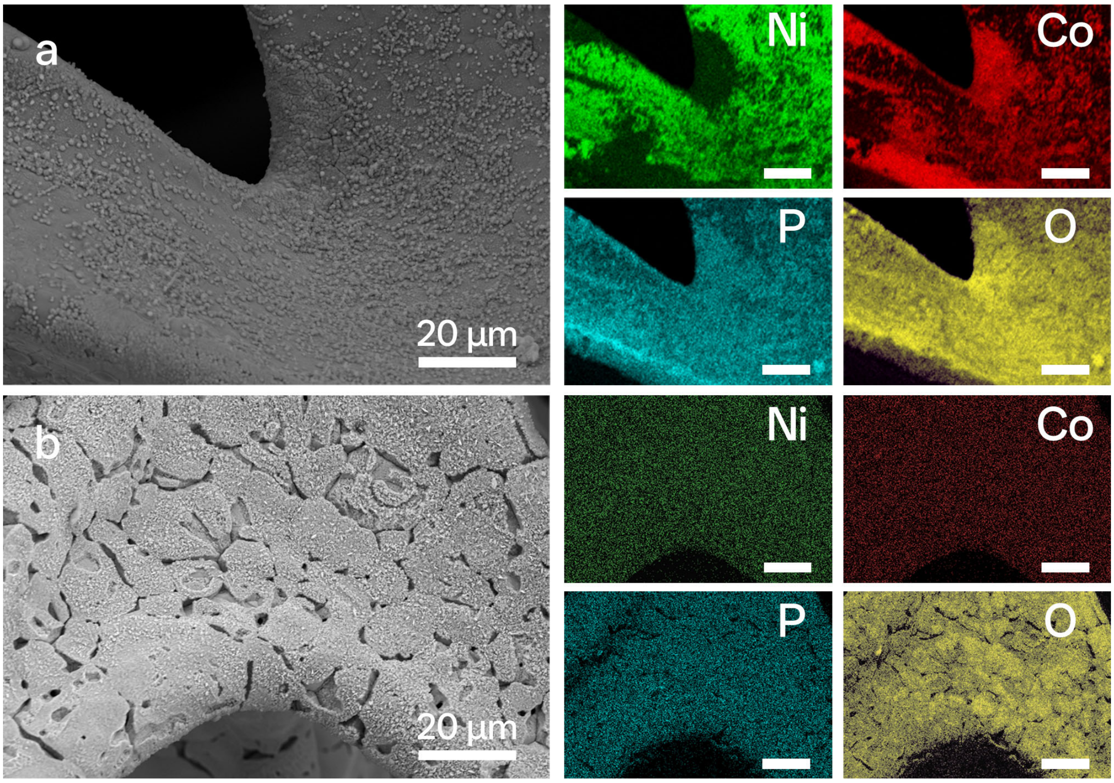

Figure S5. (a, b) SEM images and corresponding elemental distribution maps (Ni, Co, P, O) of phosphorus-doped NiCo alloy film coated on Cu foam (P-NiCo@Cu Foam), revealing homogeneous distribution of the elements. Cu foam was employed to avoid elemental interference from the Ni substrate.

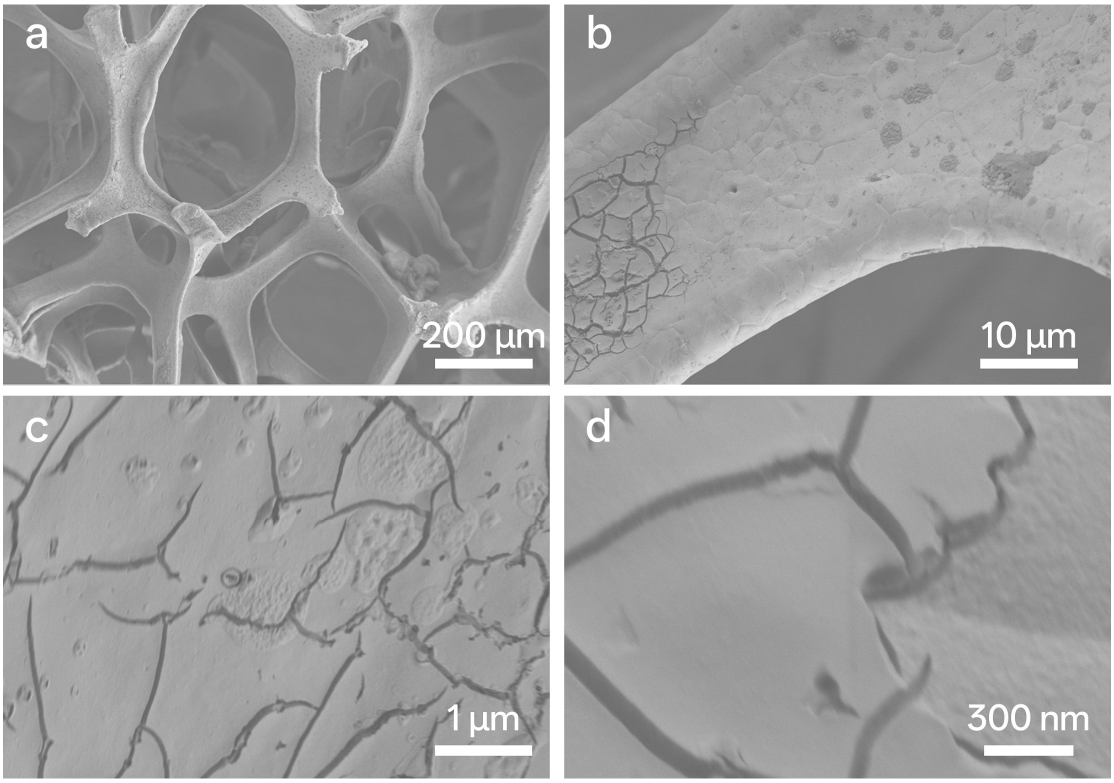

Figure S6. (a–d) SEM images of phosphorus-doped NiCo alloy film coated on Ni foam (P-NiCo@NF), showcasing surface cracks and microstructural features.

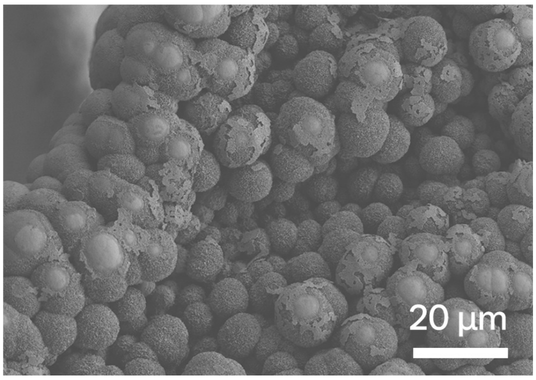

Figure S7. SEM image of hierarchical porous P-doped NiCo alloy (hpP-NiCo) synthesized via the hydrogen bubble template method, showcasing the granular hierarchical porous morphology.

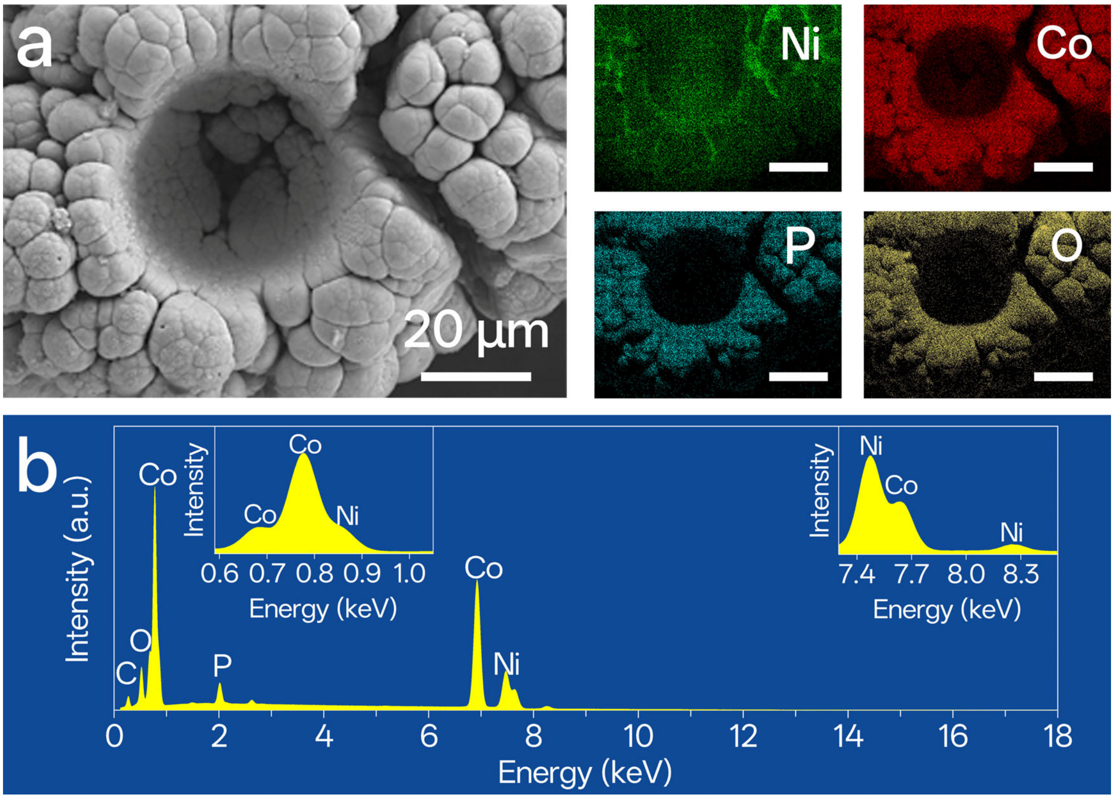

Figure S8. (a) SEM image and corresponding EDS elemental maps (Ni, Co, P, O) of hpP-NiCo, revealing homogeneous element distribution. (b) EDS spectrum of hpP-NiCo, with magnified insets displaying Co and Ni spectral regions, confirming the presence of Ni, Co, P, and O.

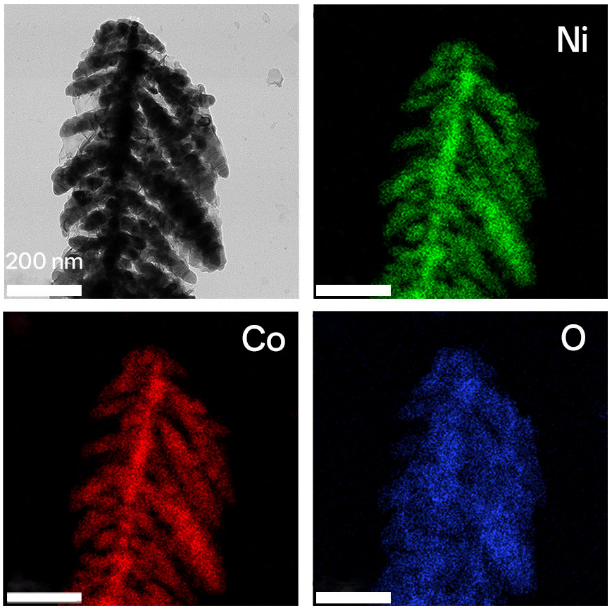

Figure S9. TEM image and corresponding EDS elemental maps (Ni, Co, O) of hierarchical porous NiCo (hp-NiCo), revealing the nanostructured dendritic architecture and uniform elemental dispersion.

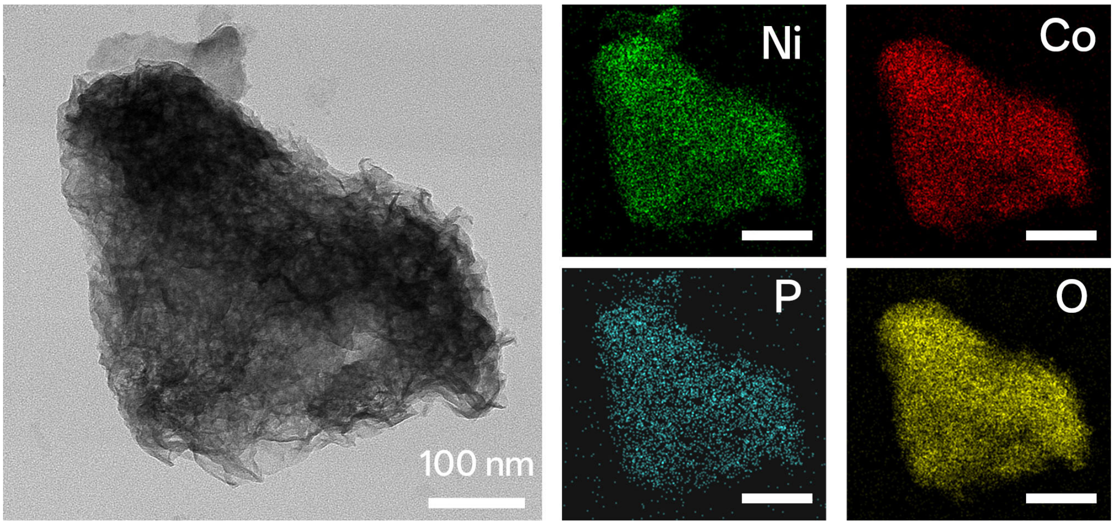

Figure S10. TEM image and corresponding EDS elemental maps (Ni, Co, P, O) of hpP-NiCo, illustrating the nanostructured porous architecture and uniform elemental dispersion at the nanoscale.

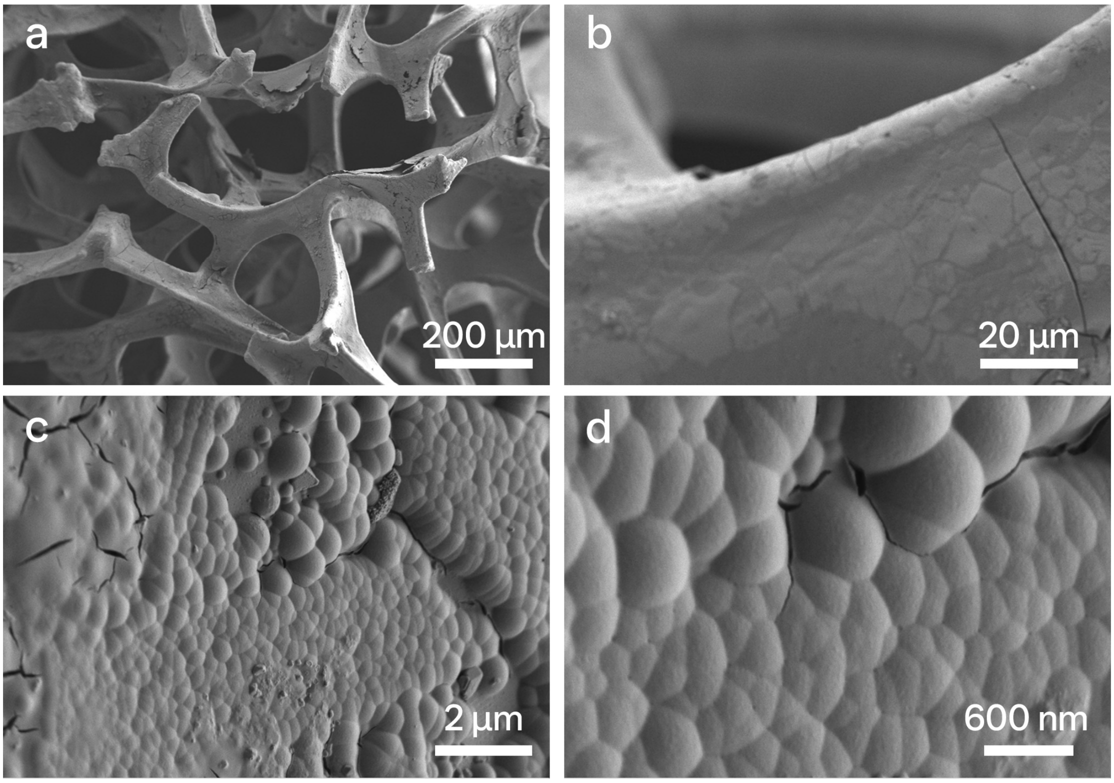

Figure S11. (a–d) SEM images of phosphorus-doped nickel film coated on Ni foam (P-Ni@NF), illustrating granular surface modification and preserved porous framework.

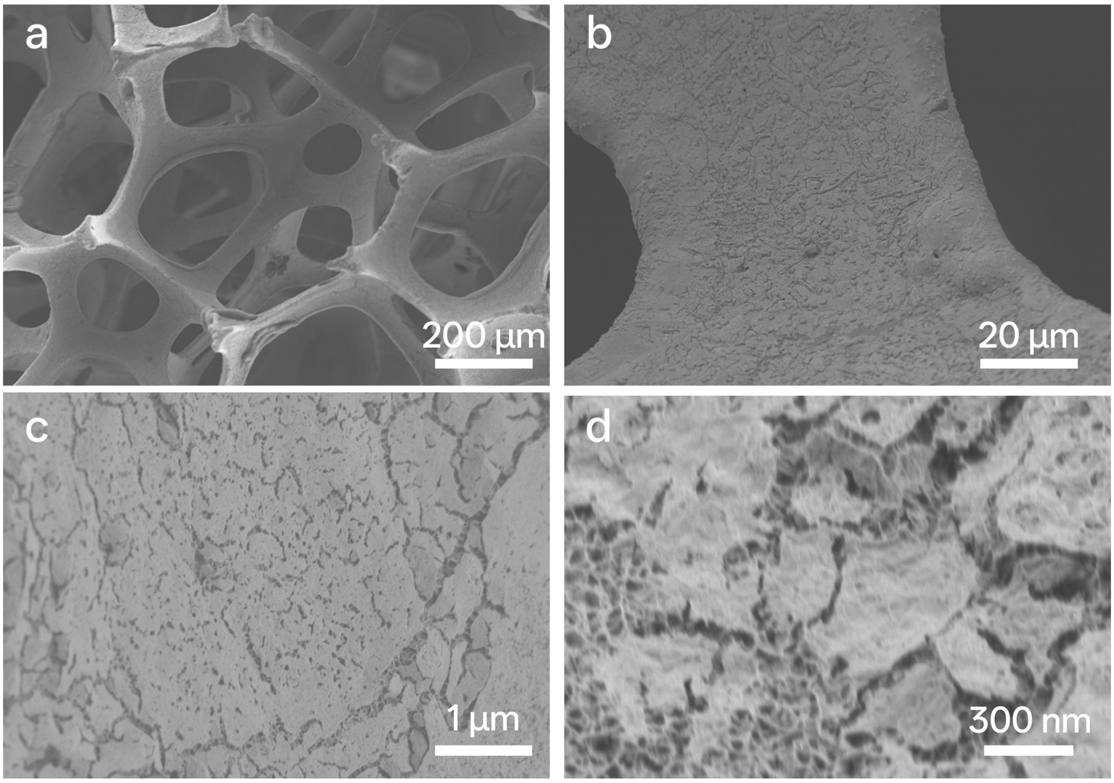

Figure S12. (a–d) SEM images of phosphorus-doped cobalt film coated on Ni foam (P-Co@NF), revealing textured surface and nanoscale heterogeneities.

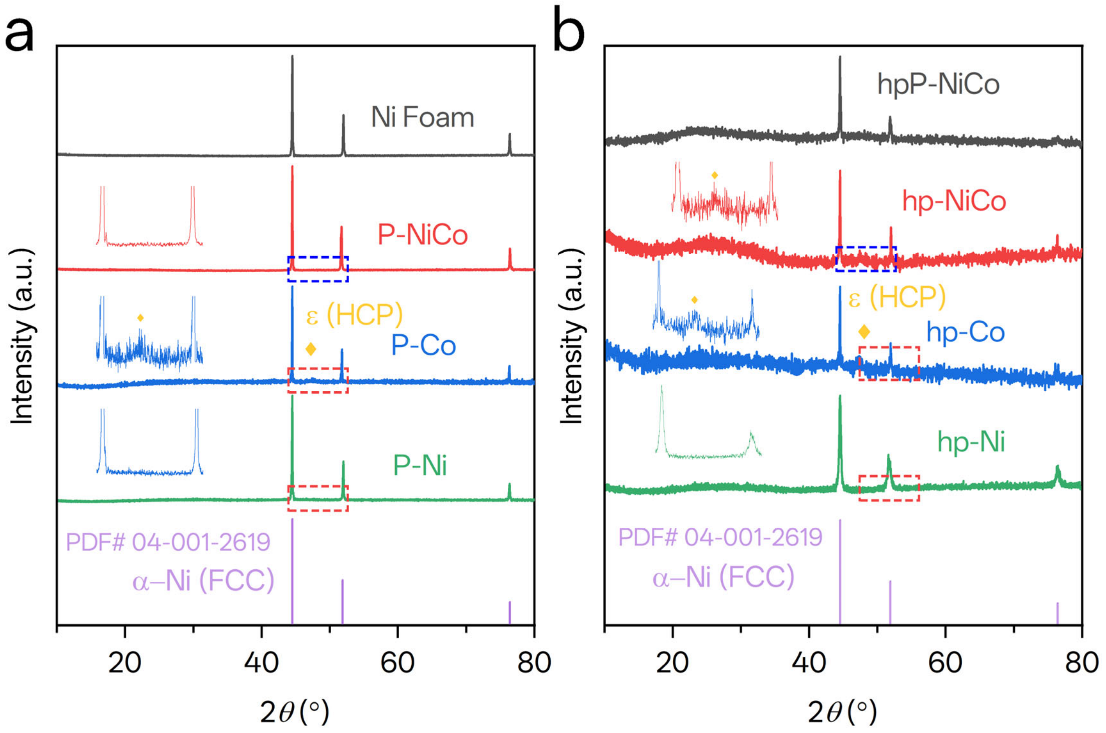

Figure S13. XRD patterns of samples deposited on Ni foam substrate. (a) Spectra of Ni foam, P-NiCo, P-Co, and P-Ni; (b) Spectra of hpP-NiCo, hp-NiCo, hp-Co, and hp-Ni. Dashed boxes with magnified insets denote regions where characteristic peaks of the ε phase (HCP) are observed in selected samples, referenced to α-Ni (FCC, PDF# 04-001-2619).

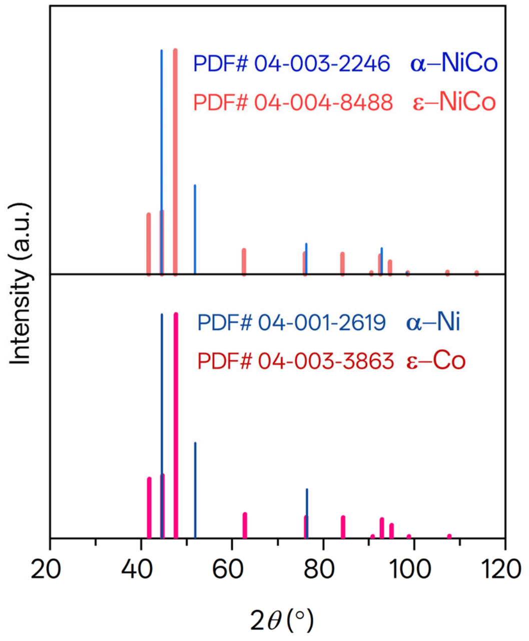

Figure S14. XRD standard patterns (PDF cards) for phase differentiation. Top panel: α-$Ni_{0.75}Co_{0.25}$ (FCC, PDF# 04-003-2246) and ε-$Ni_{0.25}Co_{0.75}$ (HCP, PDF# 04-004-8488), exhibiting distinct peak positions between the FCC and HCP phases. Bottom panel: α-Ni (FCC, PDF# 04-001-2619) and ε-Co (HCP, PDF# 04-003-3863), illustrating the close peak positions between the same phase (e.g., ε-Co vs. ε-$Ni_{0.25}Co_{0.75}$, α-Ni vs. α-$Ni_{0.75}Co_{0.25}$), which hinders phase identification.

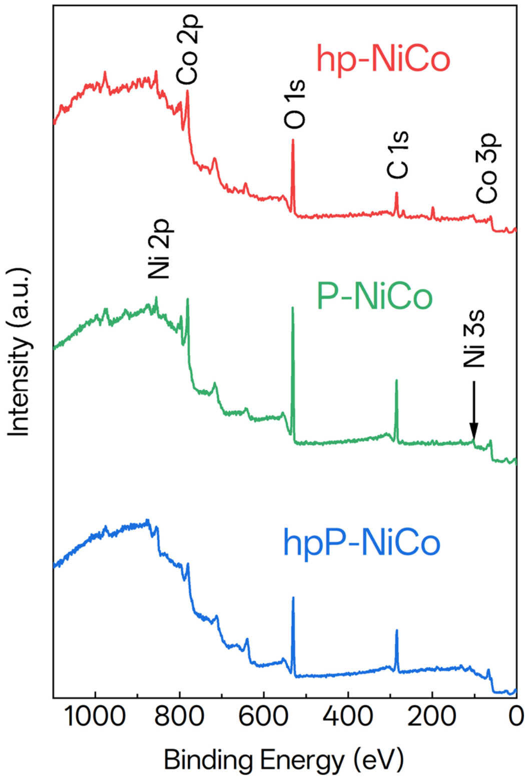

Figure S15. XPS survey spectra of hp-NiCo (red), P-NiCo (green), and hpP-NiCo (blue) exhibit characteristic peaks of Ni 2p, Co 2p, O 1s, and C 1s.

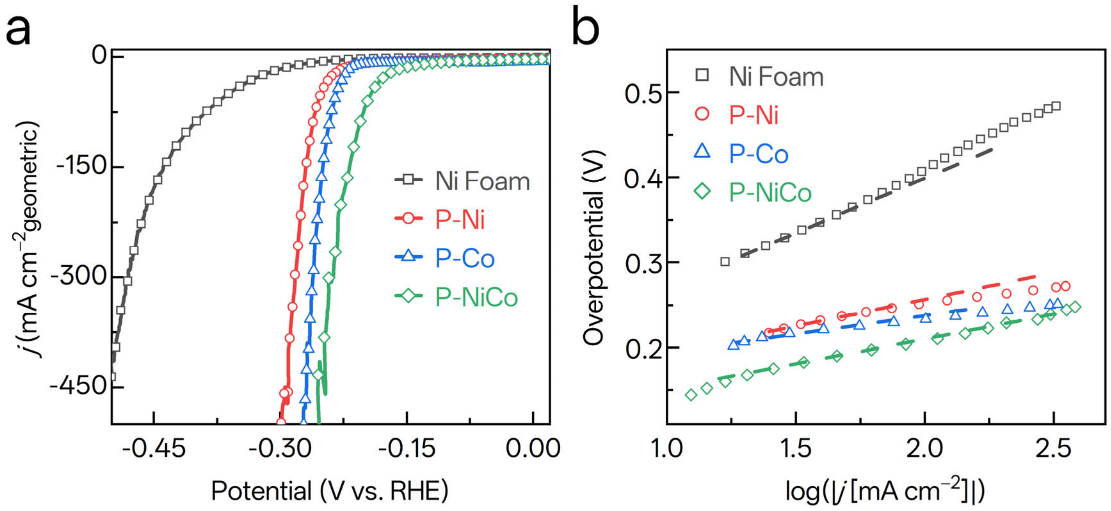

Figure S16. HER polarization curves (a) and corresponding Tafel plots (b) of Ni Foam (NF), P-Ni, P-Co, and P-NiCo under 85% *iR*-compensation, illustrating electrocatalytic activity differences.

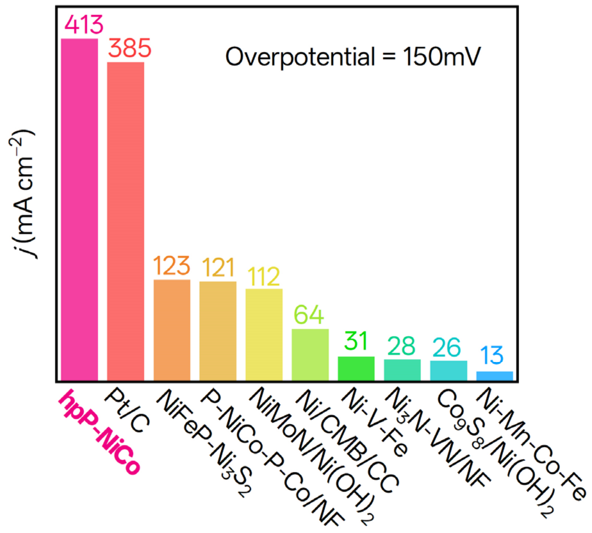

Figure S17. Bar chart comparing HER current densities at 150 mV overpotential between hpP-NiCo and reported Ni/Co-based catalysts, highlighting the superior electrocatalytic activity of hpP-NiCo.

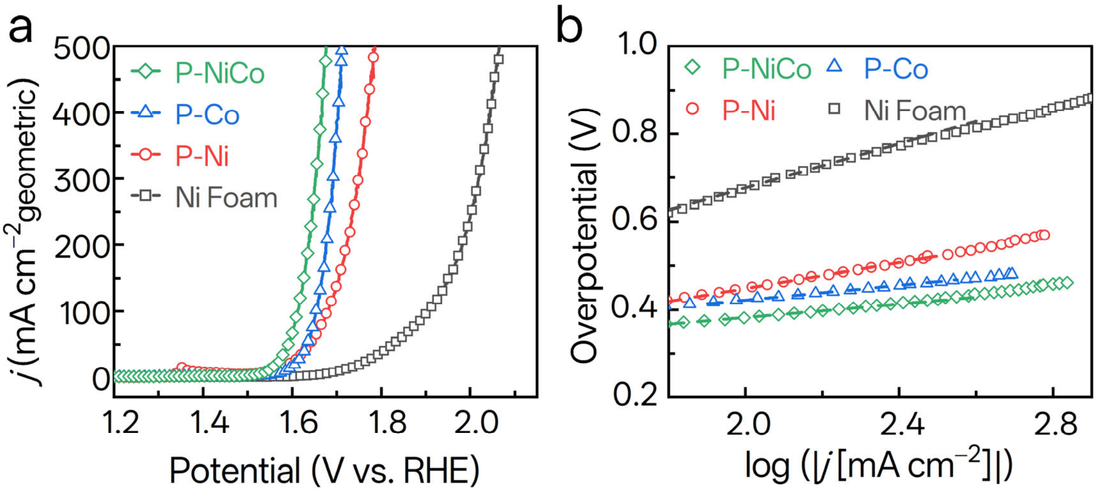

Figure S18. OER polarization curves (a) and corresponding Tafel plots (b) of Ni Foam (NF), P-Ni, P-Co, and P-NiCo under 85% *iR*-compensation, revealing kinetic and electrocatalytic activity disparities among the samples.

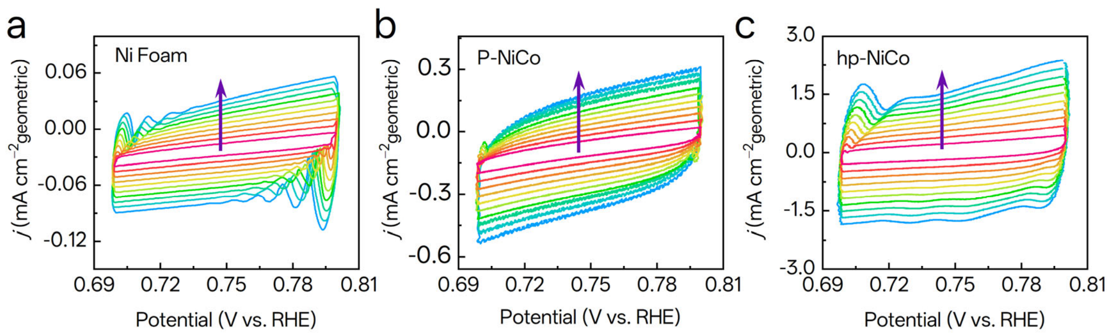

Figure S19. Cyclic voltammetry (CV) curves for capacitive current measurement at scan rates ranging from 10 to 100 mV s^{-1}, illustrating the scan rate dependence of (a) Ni foam, (b) P-NiCo, and (c) hp-NiCo catalysts.

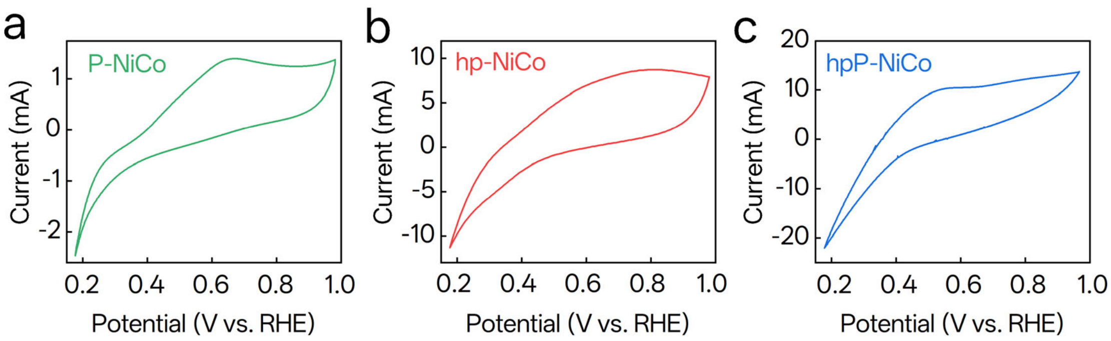

Figure S20. Cyclic voltammetry (CV) curves of (a) P-NiCo, (b) hp-NiCo, and (c) hpP-NiCo catalysts in 1 M phosphate-buffered saline (PBS) solution.

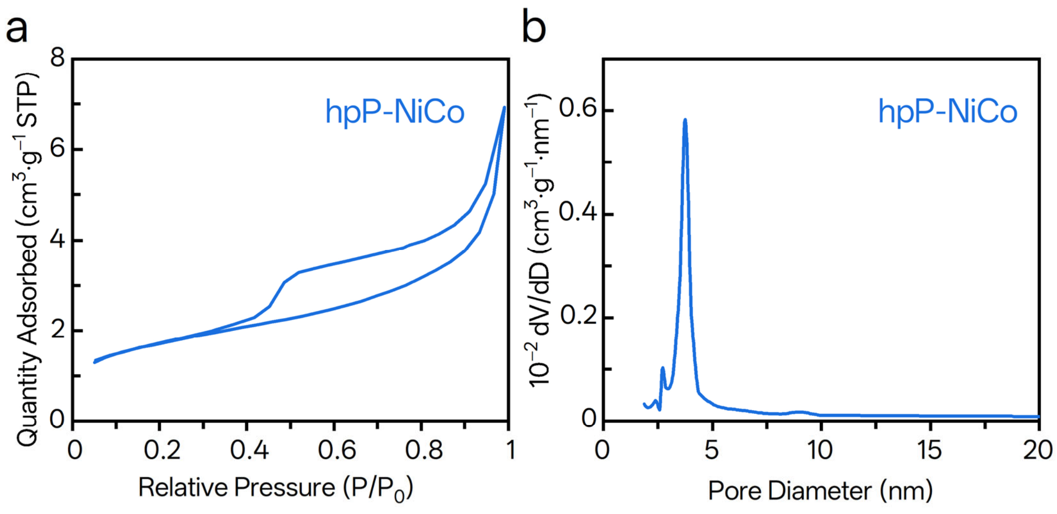

Figure S21. (a) N_2 adsorption–desorption isotherm and (b) corresponding pore size distribution curve of hpP-NiCo, characterized by Brunauer–Emmett–Teller (BET) analysis.

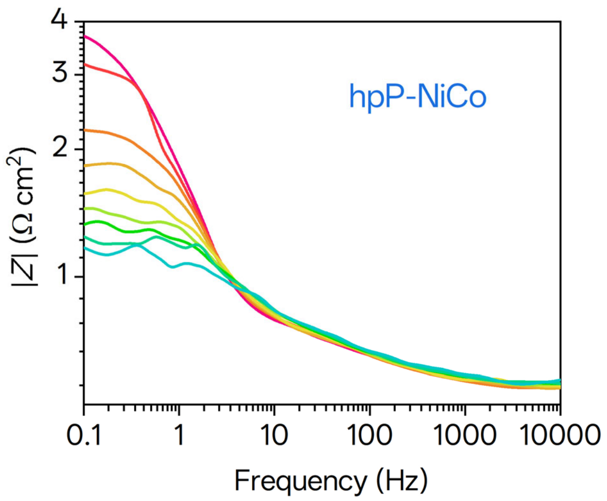

Figure S22. Bode magnitude plots of electrochemical impedance spectroscopy (EIS) for hpP-NiCo at various overpotentials, illustrating the frequency-dependent impedance response.

Table S1. Performance comparison of various HER catalysts.

Electrode	η_{100} (mV)	Ref.
Pt/C	96	This work
hpP-NiCo	119	This work
NiCoP/NCT	133	[42]
NiFeP-Ni_3S_2	152	[49]
$Ni(OH)_2/NiS_x$	180	[50]
Co(002)/CoP	183	[51]
$NiMoP_2$	199	[52]
Ni-V-Fe	218	[53]
Rh-Ni_3S_2/Co	219	[54]
Ni_2P/Ni_5P_4	270	[55]

Table S2. Performance comparison of various HER catalysts.

Electrode	j_{150} (mA cm^{-2})	Ref.
hpP-NiCo	413	This work
Pt/C	385	This work
NiFeP-Ni_3S_2	123	[49]
P-NiCo-P-Co/NF	121	[62]
NiMoN/$Ni(OH)_2$	112	[63]
Ni/CMB/CC	64	[64]
Ni-V-Fe	31	[53]
Ni_3N-VN/NF	28	[65]
Co_9S_8/$Ni(OH)_2$	26	[66]
Ni-Mn-Co-Fe	13	[67]

Table S3. Equivalent circuit fitting data of hpP-NiCo derived from electrochemical impedance spectroscopy (EIS) measurements.

η_{HER} (mV)	R_S (mΩ)	CPE1			R_{CT1} (mΩ)	CPE2			R_{CT2} (mΩ)
		Q_1	N_1	C_1 (mF)		Q_2	N_2	C_2 (mF)	
100	568	36.4	0.798	8.40	158	144	0.862	4.88	3460
110	562	60.4	0.705	42.95	173	157	0.825	8.44	2670
120	566	44.5	0.773	13.07	190	141	0.899	3.01	1540
130	564	56.3	0.729	28.99	209	143	0.916	2.46	1100
140	560	64.4	0.703	49.11	217	156	0.898	3.09	852
150	571	69.5	0.705	50.14	235	164	0.917	2.46	618
160	569	70.1	0.699	56.81	241	177	0.904	2.91	507
170	571	71.9	0.695	63.25	256	193	0.916	2.53	422
180	574	70.5	0.692	66.95	261	198	0.936	1.99	296
